# Intervention effect of physical activity on motor coordination in children and adolescents with developmental coordination disorder: a meta-analysis

**DOI:** 10.3389/fpsyg.2025.1630439

**Published:** 2025-11-19

**Authors:** Lijun Hua, Zhenpeng Song, Jiao Liu, Bin Zhang

**Affiliations:** 1 College of Physical Education, Harbin Sport University, Harbin, China; 2 Graduate School, Harbin Sport University, Harbin, China; 3 Art Institute, Wuhan Sports University, Wuhan, China

**Keywords:** physical activity, developmental coordination disorder, children and adolescents, motor coordination, meta-analysis

## Abstract

**Objectives:**

This review aims to explore the effects of physical activity on improving motor coordination in children and adolescents with developmental coordination disorders.

**Methods:**

Databases such as PubMed, Web of Science, Embase, Cochrane Library, CINAHL, and SPORTDiscus were searched according to the PRISMA framework. A total of 9 studies involving 543 participants aged 5-16 years were included. Physical activity intensity, duration of each session, weekly frequency, and total intervention cycle were set as moderating variables for subgroup analysis by Revman5.4 and Stata16 software.

**Results:**

(1) Meta-analysis showed no statistically significant improvement in motor coordination with physical activity interventions, as measured by either the MABC (MD = −0.49, 95% CI: −1.44 to 0.46, *p* = 0.31) or the MABC-2 (MD = 2.97, 95% CI: −2.25 to 8.18, *p* = 0.26) scale. (2) Subgroup and sensitivity analyses indicated notable heterogeneity across studies, which was potentially related to variations in the intervention parameters and control group conditions. While low-intensity activities appeared to show a favorable trend, this finding should be interpreted with caution, given the limited number of studies and differences in study design.

**Conclusion:**

Based on the current limited evidence, physical activity did not yield a uniform improvement in motor coordination among children and adolescents with developmental coordination disorders. However, variations in the study design, including differences in intervention intensity and control group activity, may contribute to the observed inconsistencies. Future studies with larger, well-controlled, randomized trials are needed to clarify the specific conditions under which physical activity may exert optimal benefits.

## 1 Introduction

Developmental coordination disorder (DCD) is classified as a neurodevelopmental disorder in the Diagnostic and Statistical Manual of Mental Disorders, Fifth Edition (DSM-5). DCD is a neurodevelopmental disorder primarily characterized by fine motor, gross motor, and balance disorders that cannot be explained by intellectual disabilities or other neurological diseases. The prevalence of DCD among school-age children worldwide is approximately 5–6%, with a poor prognosis for most children. Compared to healthy children, those with DCD exhibit significantly lower levels of physical activity (PA) and a higher risk of being overweight or obese (Tran et al., 2025; Hendrix et al., 2014). The symptoms often persist into adulthood (Verbecque et al., 2025; American-Psychiatric Organization, 2013; van der Hoek et al., 2012). Furthermore, DCD not only causes difficulties with basic motor skills, such as writing, tying shoelaces, and physical activities during daily life and school learning, but is also closely associated with emotional problems, reduced self-esteem, and social impairments. It exhibits a high comorbidity rate in conditions such as attention deficit hyperactivity disorder (ADHD) (Pranjić et al., 2023), severely impacting physical and mental health, as well as the social adaptation abilities of children and adolescents (Caçola, 2016).

Motor coordination is the synergy of the nervous and musculoskeletal systems. This is the basis for the development of children’s motor skills (Fernandes et al., 2016). Simultaneously, children’s motor coordination represents an integrated physical competency that synthesizes various movement abilities. It exhibits a strong correlation with other developmental capacities, particularly neurological maturation. The advancement of this ability significantly accelerates the maturation of children’s nervous systems, thereby fostering the development of language, cognitive, and emotional abilities. Long-Term Athlete Development (LTAD) identifies motor coordination as a critical developmental milestone for preschool-aged children (Lloyd et al., 2015). DCD is a motor coordination disorder that significantly impacts the daily activities and academic performance of children (Ferguson et al., 2013). Multiple studies have found that children with DCD exhibit lower PA levels compared to typical motor development. Because of poor motor coordination, they struggle to perform daily activities, leading to a lack of confidence when interacting with peers and diminishing their willingness to participate in physical activities or attempt new tasks (Hendrix et al., 2014; Li et al., 2011). Moreover, these issues become even more pronounced when children and adolescents with DCD attend public schools in low-income areas that lack the resources necessary for their development (de Villiers et al., 2012). Given this context, an accurate assessment of motor coordination in children and adolescents with DCD is fundamental to quantifying their specific functional impairments and formulating targeted intervention strategies. Among the numerous assessment tools, the Movement Assessment Battery for Children (MABC) is widely recognized for its comprehensiveness and standardization. The MABC was pioneered by Henderson and Sugden in 1992. It comprises three task dimensions: fine hand movement, positioning and grasping, and balance. By combining direct assessment with an observational scale, it enables a comprehensive evaluation of children’s motor abilities (Engel-Yeger et al., 2010). The Movement Assessment Battery for Children-Second Edition (MABC-2) further perfected age stratification and scoring systems. Validation studies have demonstrated that this scale possesses good reliability and validity (Ghayour et al., 2022). With their standardized format and widespread use, the MABC and MABC-2 provide a canonical measure of motor coordination and are used as the primary outcome measures to assess the intervention effects in this study.

PA is the process of energy expenditure caused by the contraction of skeletal muscles. Activities such as exercise, entertainment, housework, and walking for transportation can all be classified as PA. Research indicates that engaging in PA during early life stages is significantly associated with individual health (Barbosa et al., 2016). Simultaneously, numerous randomized controlled trials (RCTs), meta-analyses, and systematic reviews have demonstrated that PA improves motor skills, mental health, and cognitive function. Moreover, the effects of high-dose, high-frequency training are more pronounced (Yu et al., 2018; Alghadier and Alhusayni, 2024). Additionally, school-based Fundamental Movement Skills (FMS) training can enhance activity participation among children and adolescents with DCD and demonstrate sustained long-term effects (Sit et al., 2019). However, these studies mostly focused on aspects such as motor abilities and mental health of children and adolescents with DCD. A meta-analysis using both the MABC and MABC-2 to quantitatively summarize motor coordination abilities in children and adolescents with DCD has not been undertaken. Therefore, this study developed systematic retrieval strategies and inclusion/exclusion criteria to systematically search for studies that both implemented PA interventions for children and adolescents with DCD and utilized the MABC and MABC-2. This study aims to quantitatively assess the overall intervention effect of PA on motor coordination abilities in children and adolescents with DCD, explore the moderating effects of different PA intervention types, doses, and durations on outcome indicators, and provide evidence-based guidance for future clinical practice and high-quality research.

## 2 Methods

### 2.1 Search strategy

This study adopted the latest PRISMA 2020 guidelines for literature inclusion criteria, data organization, and statistical analysis requirements (Page et al., 2021). According to the PICOS principle, two researchers conducted a literature search in databases such as PubMed, Web of Science, Embase, Cochrane Library, CINAHL, and SPORTDiscus, utilizing a combination of subject terms and free words, to identify relevant studies. The search scope covers literature from the inception of each database to 18 August 2025. The search terms were as follows: physical activity, exercise, physical fitness, acute exercise, motor learning, neuromotor rehabilitation, children, adolescents, youth, adolescence, motor skills, childhood motor development, motor skill disorders, developmental coordination disorders, and randomized controlled trials. All retrieved documents were imported into EndNote software for duplication reduction. Subsequently, two researchers independently screened the literature. In cases of disagreement, a third researcher was consulted for resolution.

### 2.2 Study selection

The criteria for screening, inclusion, and exclusion of studies were developed based on PICOS principles. The literature inclusion criteria were as follows: (1) The type of experiment was an RCT. (2) The study subjects were children and adolescents with DCD aged 5–18 years. (3) The intervention included various types of PA, including sports, play, and fitness activities. (4) The outcome indicators employed were MABC and MABC-2.

The literature exclusion criteria were as follows: (1) Experimental participants were adults. (2) Studies in which the intervention did not involve PA. (3) Studies lacking a control group. (4) Studies in which the necessary data could not be extracted even after contacting the corresponding authors. (5) Studies that did not employ a pre-test–post-test design with both experimental and control groups. (6) Qualitative research, reviews, non-intervention studies, dissertations, and conference papers.

### 2.3 Data extraction and quality assessment

Data extraction was performed independently by two researchers using Excel software based on the inclusion and exclusion criteria. In case of disagreement, consensus was reached through discussion and consultation with a third researcher. This study included the following information: (a) Basic characteristics of the included subjects (first author, publication year, number of participants in the experiment, country, gender in the experiment, diagnostic criteria for DCD, presence of complications); (b) PA variables (type of PA, PA intensity, weekly frequency, session duration, and total intervention cycle); and (c) Pre-test–post-test data of outcome indicators (MABC and MABC-2).

The quality of the literature was assessed independently by two researchers using the Cochrane Risk of Bias Assessment Tool and the PEDro scale. In cases of disagreement between the two researchers, a third researcher was consulted for quality assessment. The Cochrane Risk of Bias Assessment Tool evaluates the following aspects: whether the use of randomization methods is clearly described, whether allocation was concealed, whether blinding was implemented for investigators or patients and outcomes, whether selective reporting bias existed, whether outcome data were complete, and whether other biases were present. Based on these criteria, the studies included in the literature were categorized as having a high, low, or unclear risk of bias. The specific content of each PEDro scale item included (a) clear inclusion criteria, (b) random assignment, (c) allocation concealment, (d) similar baseline characteristics, (e) participant blinding, (f) clinician blinding, (g) assessor blinding, (h) adequate follow-up (85% or more of participants completing the trial), (i) intention-to-treat analysis, (j) between-group comparisons, and (k) point estimates and variability. Scoring criteria: Yes = 1 point; No = 0 points. The first criterion was not scored: 9–10 points indicated extremely high quality, 6–8 points indicated high quality, 4–5 points indicated medium quality, and ≤3 points indicated low quality.

### 2.4 Statistical analyses

Quantitative analysis of the included studies’ data was performed using RevMan 5.4 and Stata 16 software. Within the two outcome measures of MABC and MABC-2, the included studies used a unified assessment scale, so the mean difference (MD) and its 95% confidence interval (CI) were used to report effect sizes. Heterogeneity was assessed using *I*^2^. When *I*^2^ ≤ 50% and *p* ≥ 0.1, small heterogeneity was indicated, warranting a fixed-effects model. When *I*^2^ was > 50% and *P* was < 0.1, small heterogeneity was present, necessitating a random-effects model. Additionally, sensitivity and subgroup analyses should be conducted to explore the sources of heterogeneity (Mavridis and Salanti, 2014; Song et al., 2001). Significance level *α* = 0.05. Funnel plots and Egger’s test were used to assess the risk of publication bias. If a high risk of publication bias was present, trimming methods were used to evaluate the impact. Specifically, RevMan 5.4 software was used for forest plot generation, quality assessment, and subgroup analysis. Stata 16 software was used for sensitivity analysis and Egger’s test.

## 3 Results

### 3.1 Literature selection

**Figure 1:**
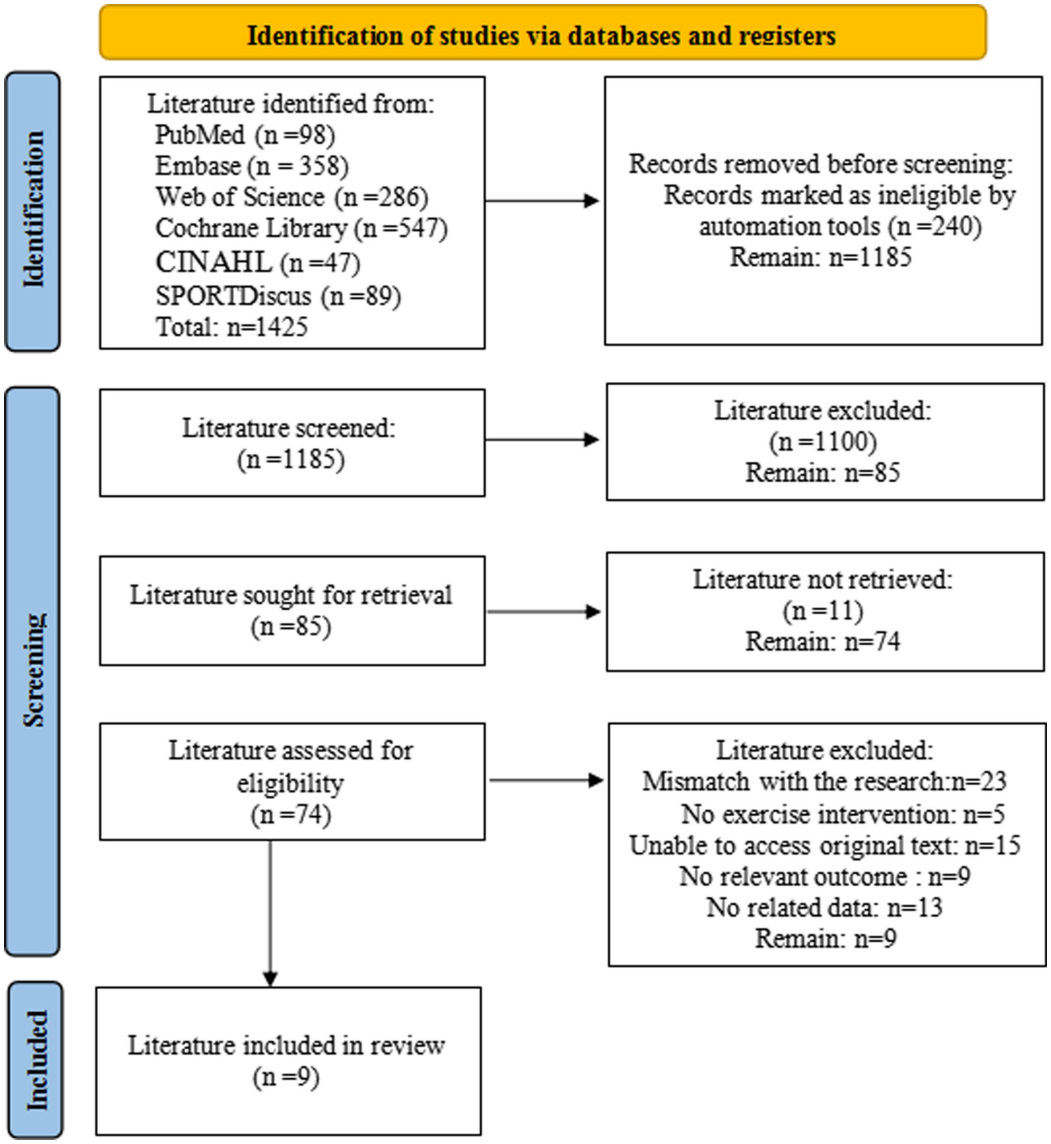
Flowchart.

According to the search strategy, 1,425 articles were retrieved from all databases. After removing 240 duplicate papers using EndNote literature management software, titles and abstracts were screened by two authors. A consensus should be reached between the two. In the case of any remaining disagreements, a third reviewer was consulted. Approximately 1,100 documents were eliminated after reading, and 76 were eliminated after reading the full text. Finally, nine studies included could be meta-analyzed. The literature screening process is shown in Figure 1.

### 3.2 Study characteristics

Table 1 shows the main characteristics of all included studies. All nine studies were RCTs that incorporated pre- and post-tests. These nine studies included 543 children and adolescents with DCD. The smallest sample size was 12 participants, while the largest was 145. One study recruited only female participants, and one did not specify the gender of the subjects. The publication dates of these studies ranged from 2010 to 2024, with participants aged 5–16. Regarding the types of PA, five studies employed selective exercises; one study utilized a multi-group experimental design with three intervention groups: Tai Chi plus strength training, Tai Chi alone, and strength training alone; one study adopted a group exercise mode; one study implemented task-specific balance training; one study incorporated Taekwondo training; and one study utilized task-oriented functional training. Regarding PA intensity, two studies were low-intensity, five studies were moderate-intensity, and two studies were vigorous-intensity. In terms of weekly frequency, six studies involved exercising once per week, two studies involved exercising twice per week, and one study involved exercising three times per week. Session duration varied across the included studies: one study involved a 30-min session, two studies involved 45-min sessions, four studies involved 60-min sessions, and two studies involved 90-min sessions. The total intervention cycle varied across the included studies: two studies had cycles of 5 and 6 weeks, respectively; two studies had a cycle of 8 weeks; four studies had a cycle of 12 weeks; and one study had a cycle of 14 weeks. All studies used the MABC test to assess motor coordination in children and adolescents with DCD. Among these, five studies utilized the MABC scale, whereas four studies employed the MABC-2 scale.

**Table 1:** Basic characteristics of the included studies.

Author (Year)	Nationality	Age	*N*	*n*		Diagnostic criteria	Symptoms	Type	Control group	Intensity	Weekly frequency	Session duration	Total intervention cycle	Outcome indicators
				Female	Male									
Wilson et al. (2016)	Canada	7–12 years old	24	–	–	DSM-5	NO	Selective practice	No exercise	Low intensity	1 time	60 min	5 weeks	MABC
Hillier et al. (2010)	Australia	5–8 years old	12	2	10	DSM-IV	NO	Selective practice	No exercise	Low intensity	1 time	30 min	6 weeks	MABC
Lee (2024)	Korea	8–9 years old	55	20	35	DSM-5	NO	Selective practice	physical education	Moderate intensity	3 times	60 min	12 weeks	MABC-2
Cavalcante et al. (2020)	Brazil	7–10 years old	32	8	24	DCDQ, DSM-5	NO	Selective practice	Wii tasks	Moderate intensity	2 times	60 min	8 weeks	MABC-2
Fong et al. (2022)	China	9–12 years old	121	20	101	DSM-5	NO	(1) TC-MPT (2) TC (3) MPT	Usual treatment	Moderate intensity	1 time	90 min	12 weeks	MABC-2
Hung and Pang (2010)	China	6–10 years old	23	4	19	DSM-IV-TR	NO	Selective practice	individual sports	Moderate intensity	1 time	45 min	8 weeks	MABC
Ma et al. (2018)	China	6–9 years old	145	24	121	DSM-5-TR	NO	Taekwondo	jogging	Vigorous intensity	1 time	60 min	12 weeks	MABC
Fong et al. (2016)	China	7–8 years old	88	27	61	DSM-IV	NO	Task-specific balance training	No exercise	Moderate intensity	2 times	90 min	12 weeks	MABC
Bonney et al. (2017)	South Africa	13–16 years old	43	43	0	DSM-5	NO	Task-oriented Functional Training	Wii training	Vigorous intensity	1 time	45 min	14 weeks	MABC-2

*N* is the total sample size; *n* is the sample size; MABC: Standardized Assessment of Children’s Motor Coordination Test; MABC-2: Standardized Assessment of Children’s Motor Coordination Test (Second Edition).

### 3.3 Quality assessment

**Figure 2:**
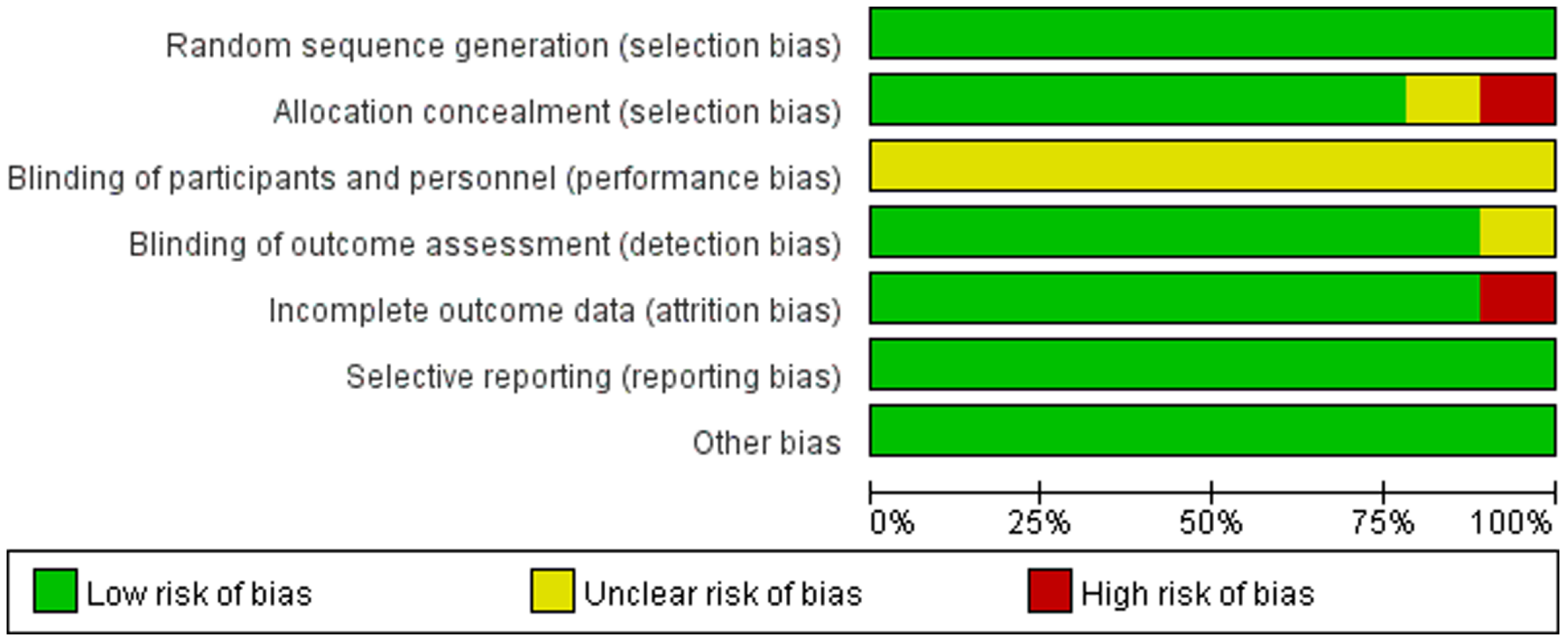
Percentage of bias risk items included in the study.

Quality assessment of the included studies was conducted using the Cochrane Risk of Bias Tool. The overall risk of bias results is shown in Figure 2.

Quality assessment of the included studies was conducted using the PEDro scale. All studies adhered to the principles of randomization, baseline similarity, statistical analysis of intergroup results, point measurement, and calculation of difference values. Six studies used allocation concealment. Blinding was performed on outcome assessors in eight studies. PEDro scores ranged from 6 to 8, indicating an overall high quality (Table 2).

**Table 2:** PEDro scores of the included studies.

Study	Item 1	Item 2	Item 3	Item 4	Item 5	Item 6	Item 7	Item 8	Item 9	Item 10	Item 11	Score	Quality
Wilson et al. (2016)	√	√		√			√	√	√	√	√	7	High
Hillier et al. (2010)	√	√	√	√			√	√	√	√	√	8	High
Lee (2024)	√	√		√				√	√	√	√	6	High
Cavalcante et al. (2020)	√	√		√			√	√	√	√	√	7	High
Fong et al. (2022)	√	√	√	√			√		√	√	√	7	High
Hung and Pang (2010)	√	√	√	√			√	√	√	√	√	8	High
Ma et al. (2018)	√	√	√	√			√		√	√	√	7	High
Fong et al. (2016)	√	√	√	√			√	√	√	√	√	8	High
Bonney et al. (2017)	√	√	√	√			√	√	√	√	√	8	High

### 3.4 Meta-analysis results

#### 3.4.1 Effects of PA on the MABC scale in children and adolescents with DCD

**Figure 3:**
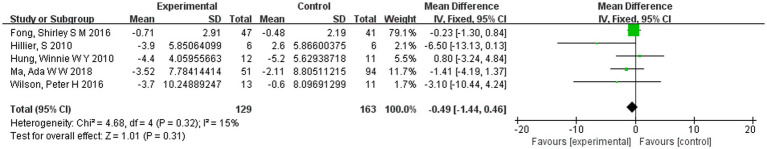
Forest plot of the effect of PA on the MABC scale in children and adolescents with DCD.

For the MABC scale, 5 studies (Wilson et al., 2016; Hillier et al., 2010; Fong et al., 2022; Hung and Pang, 2010; Ma et al., 2018) were included in the literature, with a total sample size of 292 subjects. Since the same scoring criteria for the MABC scale were used across studies, the MD was selected for effect size pooling. The results showed no significant heterogeneity (*I^2^* = 15%, *p* = 0.32); thus, a fixed-effects model was applied for the analysis. The combined effect size was MD = −0.49, Z = 1.01 (*p* = 0.31) with a 95% CI of −1.44 and 0.46. The forest plot (Figure 3) shows that the MD 95% CI for the effect of PA on children’s motor coordination development aligns with the line of no effect. This indicates that there was no statistically significant difference between the PA and control groups in the MABC outcome indicators for children and adolescents with DCD (*p* = 0.31). Although the meta-analysis revealed an overall negative effect with low heterogeneity among the studies (*I*^2^ = 15%), we conducted exploratory subgroup analyses to investigate whether the effects of PA interventions were influenced by key covariates to gain deeper insights into these findings. Given the limited number of included studies (*n* = 5), three covariates were selected for analysis: PA intensity, session duration, and total intervention cycle. Each variable was divided into two subgroups based on statistical characteristics, ensuring that each subgroup contained at least two studies. The results of the subgroup analysis are summarized in Table 3, and the corresponding forest plots are shown in Supplementary Figure S1.

**Table 3:** Subgroup analysis of the effect of PA on the MABC scale in children and adolescents with DCD.

Regulated variable	Heterogeneity				Subgroup	*N*	MD	[95%CI]	Two-tailed test	
	*p*	*I* ^2^	*p*	Differences					*Z*	*p*
Intensity	0.50	0%	0.07	69.80%	Low intensity	2	−4.97	[−9.89, −0.05]	1.98	0.05
	0.63	0%			Moderate to vigorous intensity	3	−0.31	[−1.28, 0.65]	0.64	0.53
Session duration	0.57	0%	0.68	0%	≥60 min	3	−0.43	[−1.42, 0.56]	0.85	0.39
	0.07	71%			<60 min	2	−1.18	[−4.63, 2.27]	0.67	0.50
Total intervention cycle	0.16	45%	0.49	0%	≤8 weeks	3	−1.53	[−4.65, 1.60]	0.96	0.34
	0.44	0%			>8 weeks	2	−0.38	[−1.38, 0.62]	0.75	0.45

*p* < 0.05 Means a statistically significant difference, and *p* < 0.01 means a statistically significant difference.

Although the overall pooled analysis showed no statistically significant effect of the intervention, a clear trend was observed in the PA intensity subgroup analysis based on our pre-specified subgroup analyses. The effect size was relatively larger in the low-intensity PA intervention group (MD = −4.97, 95% CI: −9.89, −0.05; *p* = 0.05), approaching statistical significance. In contrast, the effect size was smaller and non-significant in the moderate-to-vigorous-intensity PA group (MD = −0.31, 95% CI: −1.28, 0.65; *p* = 0.53). The confidence intervals for the two sets of effect values did not overlap. The *p*-value of the between-group differences was 0.07, failing to reach statistical significance. However, the heterogeneity index (*I*^2^ = 69.8%) indicated that PA intensity was likely the main factor contributing to the differences among studies. In contrast, subgroup analyses based on session duration (≥60 min and <60 min) and total intervention cycle (≤8 weeks and >8 weeks) revealed no statistically significant differences in effect sizes between subgroups (between-group *p* values of 0.68 and 0.49, respectively), with low between-group heterogeneity (*I*^2^ = 0%). Furthermore, the pooled effect sizes within each subgroup failed to demonstrate statistical significance.

#### 3.4.2 Effects of PA on the MABC-2 scale in children and adolescents with DCD

**Figure 4:**
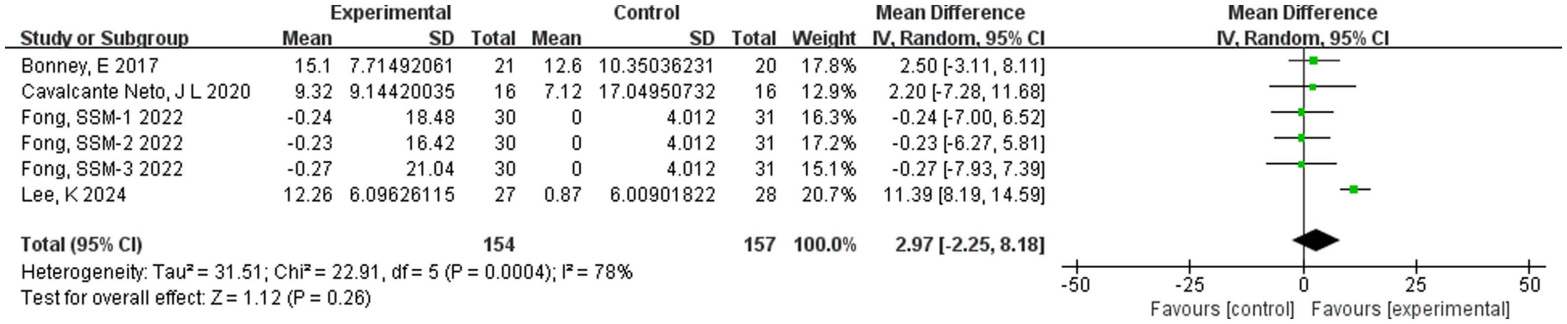
Forest plot of the effect of PA on the MABC-2 scale in children and adolescents with DCD.

For the MABC-2 scale, 4 studies (Lee, 2024; Cavalcante et al., 2020; Fong et al., 2016; Bonney et al., 2017) were included in the literature, and the sample size of subjects was 251. Since the MABC-2 scoring criteria were consistent across studies, MD was selected for effect size pooling. The results exhibited significant heterogeneity (*I*^2^ = 78%, *p* < 0.01), and the random-effects model was used for the analysis. The combined effect size is MD = 2.97, Z = 1.12 (*p* = 0.26) with a 95% CI of −2.25 and 8.18. The forest plot (Figure 4) shows that the MD 95% CI for the effect of PA on children’s motor coordination development aligns with the line of no effect. This indicates that there was no statistically significant difference between the PA and control groups in the MABC-2 outcome indicators for children and adolescents with DCD (*p* = 0.26). Although the meta-analysis revealed an overall negative effect, the studies exhibited high heterogeneity (*I*^2^ = 78%). To further investigate the sources of heterogeneity, we conducted subgroup analyses. Given the limited number of included studies (*n* = 4), we selected three covariates for analysis: PA intensity, session duration, and weekly frequency. Each covariate was divided into two subgroups based on statistical characteristics, ensuring that each subgroup contained at least two studies. The results of the subgroup analysis are summarized in Table 4, and the corresponding forest plot is shown in Supplementary Figure S2.

**Table 4:** Subgroup analysis of the effect of PA on the MABC-2 scale in children and adolescents with DCD.

Regulated variable	Heterogeneity				Subgroup	*N*	MD	[95%CI]	Two-tailed test	
	*p*	*I* ^2^	*p*	Differences					*Z*	*p*
Intensity	0.78	0%	0.41	0%	Vigorous intensity	3	0.98	[−2.78, 4.75]	0.51	0.61
	0.00	84%			Low to moderate intensity	3	4.93	[−3.68, 13.53]	1.12	0.26
Session duration	0.01	78%	0.13	57.40%	≤60 min	3	6.05	[−1.00, 13.09]	1.68	0.09
	1.00	0%			>60 min	3	−0.24	[−4.13, 3.64]	0.12	0.90
Weekly frequency	0.07	69%	0.12	57.50%	<2 times	2	7.92	[−0.81, 16.65]	1.78	0.08
	0.89	0%			≥2 times	4	0.65	[−2.55, 3.84]	0.40	0.69

*p* < 0.05 Means a statistically significant difference, and *p* < 0.01 means a statistically significant difference.

The results indicate that none of the examined covariates can effectively explain the sources of heterogeneity across studies. Specifically, within the different PA intensity subgroups, vigorous-intensity (*I*^2^ = 0%) and low-to-moderate-intensity (*I*^2^ = 84%) showed no statistically significant differences in effect sizes between subgroups (*p* = 0.41). In the different session duration subgroups, the ≤60-min group (*I*^2^ = 78%) and the >60-min group (*I*^2^ = 0%) were not statistically significant in effect size between subgroups (*p* = 0.13). There was no significant difference in effect size between subgroups <2 times (*I*^2^ = 69%) and ≥2 times (*I*^2^ = 0%) in the subgroups with different weekly PA frequencies (*p* = 0.12). Meanwhile, our subgroup analysis showed that no subgroup classification exhibited insignificant heterogeneity within groups, which subsequently became significant after pooling. It is noteworthy that after subgroup analysis, significant heterogeneity persisted within multiple subgroups, suggesting that more important unidentified factors have driven the variation in outcomes.

### 3.5 Publication bias test and heterogeneity analysis

Given that this meta-analysis included only five independent studies on MABC outcomes and four independent studies on MABC-2 outcomes, traditional quantitative methods for detecting publication bias cannot guarantee statistical reliability. Egger’s test relies on the statistical efficacy of the regression model; its efficacy decreases dramatically when there are fewer than 10 articles, and it is prone to produce false-negative or false-positive conclusions and thus is not suitable for use (Sterne and Egger, 2006; Macaskill et al., 2001). Second, although funnel plots can be drawn to visualize the symmetry of the distribution of effect sizes, they are highly sensitive to a single extreme effect point when the number of studies is very small, and the plots themselves are too unstable to provide solid evidence for publication bias (Valentine et al., 2011; Ioannidis and Trikalinos, 2007). Based on this, a combination of the quantitative Egger’s test and qualitative description was applied for judging publication bias in this study. The Egger’s test was first used to test for publication bias. The results showed no statistically significant difference between the total score of the MABC scale (*t* = −0.94, *p* = 0.416) and the MABC-2 scale (*t* = 1.25, *p* = 0.278), indicating that there was no publication bias (Supplementary Table S1; Supplementary Figures S3, S4). In addition, this study qualitatively assessed publication bias from a systematic traceability and small-sample effect perspective. All 9 studies for outcome indicators were published in peer-reviewed journals, and no gray literature or unpublished results were retrieved. This study suggests that the sample size should be expanded in the future to validate the current findings because of the small amount of literature.

The above analysis found that there was high heterogeneity in the MABC-2 indicators. Sensitivity analyses were performed on four MABC-2 studies using a study-by-study exclusion method to test whether the heterogeneity originated from specific literature. The results found a combined MD of 2.97 (95% CI: −2.25, 8.18; *I*^2^ = 78%) when the MABC-2 outcome metric was included in all 4 studies. Heterogeneity was driven primarily by the study of Lee (2024) after study-by-study exclusion (*I*^2^ = 0% after exclusion, MD = 0.80, 95% CI: −2.22, 3.83). Upon thorough verification, we found that the control group in Lee (2024) utilized only routine PA, whereas the control groups in the other studies incorporated more targeted training (such as perceptual-motor training and conventional physical therapy). This significant difference in the nature of the interventions in the control groups is likely the primary reason for the divergence of the results from those of other studies, thereby leading to high heterogeneity. After excluding this study, the remaining studies exhibited greater homogeneity in their control group protocols, and the results became more stable. However, due to insufficient sample size, further subgroup analysis could not be conducted. Therefore, these studies were retained for inclusion.

## 4 Discussion

This study systematically evaluated the effects of PA on motor coordination in children and adolescents with DCD using a meta-analysis. The results indicated that the overall combined effect size of the intervention did not reach statistical significance when the MABC or MABC-2 scale was used as the assessment tool. This finding differs from those of some previous studies, which may be attributed to the limited number of studies included in this meta-analysis and the high methodological heterogeneity, making it difficult to draw a unified and definitive conclusion (Alghadier and Alhusayni, 2024). However, a deeper analysis reveals that the heterogeneous patterns exhibited by the MABC and MABC-2 scales hold significant implications. In the background of the low overall heterogeneity of the MABC scale, our subgroup analysis indicates that PA intensity may serve as a key moderating factor. Regarding the MABC-2 scale, although subgroup analysis has not yet identified the source of its heterogeneity, sensitivity analysis suggests that its heterogeneity may stem from substantial differences in the intervention protocols of the control group.

Analysis of the MABC scale provided insightful findings. Although the overall effect was not significant, subgroup analysis based on PA intensity revealed that the result in the low-intensity group approached significance (MD = −4.97, 95% CI: −9.89, −0.05, *p* = 0.05), with a larger effect size than that in the moderate-to-vigorous-intensity group (MD = −0.31, 95% CI: −1.28, 0.65, *p* = 0.53). The lack of a universally accepted minimum clinically important difference (MCID) for the MABC scale in this field limits the precise clinical interpretation of a − 4.97-point improvement. This value itself holds significant implications. The MABC score can assess overall motor ability in multiple dimensions (manual dexterity, ball skills, and balance), and its change often indicates progress in multiple daily tasks. In comparison, the pooled analysis of the MABC-2 scale also failed to reveal statistically significant differences (MD = 2.97, 95% CI: −2.25, 8.18, *p* = 0.26), with substantial heterogeneity among the studies (*I*^2^ = 78%). Further subgroup analysis failed to effectively explain this discrepancy, but sensitivity analyses revealed that the Lee (2024) study, which employed only routine PA as its control group, differed markedly from the more targeted intervention protocols in other studies, thereby emerging as the primary source of heterogeneity. It is suggested that future studies should place greater emphasis on the scientific rigor and comparability of control conditions to avoid masking intervention effects due to control group differences. Furthermore, the broader age range (3–16 years) and more complex task dimensions of the MABC-2 scale amplified subtle differences in population targeting and assessment focus across various studies. Therefore, the considerable heterogeneity in MABC-2 results may stem from differences in study protocols and sample characteristics rather than methodological bias alone. Across the included trials, the control group activities ranged from no exercise to general physical education or other low-engagement tasks. Such variability may have influenced the between-group contrasts and may partly explain the apparent differences observed between the intensity subgroups. This finding highlights the need for more consistent and clearly defined control conditions in future randomized trials. Instead, it should be interpreted as follows: Under the current evidence base, intervention outcomes are influenced by multiple factors, including study design, control protocols, and population characteristics, making it impossible to draw a simple, unified conclusion. Clinically, although the overall results for MABC-2 did not reach statistical significance, the proposed MCID (MCID ≈ 1.39 points, sensitivity 72.5%, specificity 46.2%) for the MABC-2 scale, based on existing literature, still provides a reference for interpreting results (Griffiths et al., 2018). It should be emphasized that this MCID is derived from studies of moderate methodological quality and should be treated with caution. However, we observe that the mean change in some subgroups of this study exceeded this threshold when using this threshold as a rough reference. This suggests that even when statistical significance is not achieved, the magnitude of improvement may still hold practical significance for the motor coordination function of children and adolescents with DCD. In other words, the potential value of PA interventions may be obscured by limited sample sizes and methodological differences; however, this still holds positive implications for clinical and educational practice.

Although existing research has confirmed that school-based (Sit et al., 2019) or family based PA programs (Harkness-Armstrong et al., 2025), as well as comprehensive intervention protocols (Moon et al., 2024), are generally effective in improving motor skills among children and adolescents with DCD, there remains no consensus on which intervention parameters are the most critical. Our exploratory subgroup analysis tentatively suggests that PA intensity may influence intervention outcomes. However, given the small number of studies and the confounding effects of variability in the control group, this interpretation remains provisional and requires validation in larger, well-controlled trials. If this trend is confirmed, the potential superiority of low-intensity PA could be explored through several neurobiological and cognitive frameworks. Based on previous studies, low-load, high-repetition training may have lower requirements for the neural circuits of children with DCD. Neuroimaging evidence showed that children with DCD often exhibit compensatory prefrontal overactivation during motor tasks, indicating that they require more cognitive effort to achieve motor control (Deng et al., 2014). Therefore, we can infer that lower-intensity activities might reduce this cognitive load and facilitate motor learning efficiency. A similar trend was observed in a study of the dose–response effects of exercise on inhibitory control in children with ADHD, where low-to-moderate-intensity exercise yielded greater benefits for inhibitory control than high-intensity exercise (Tsai et al., 2021). At the neurophysiological level, it is well established that PA promotes neuroplasticity. Exercise can upregulate factors, such as BDNF, which support synaptic plasticity (de Sousa et al., 2020), and animal studies show that it can increase dendritic spine density in motor areas (Khalki et al., 2024), providing a general foundation for how PAs might support neural adaptation. However, the specific mechanistic links to PA intensity in children and adolescents with DCD remain speculative. The primary value of our subgroup findings is that they highlight the need for future research. It is hoped that future studies will directly test these hypotheses and determine whether PA intensity is a genuine moderator of intervention effects.

In summary, while this meta-analysis did not confirm a universal improvement in motor coordination among children and adolescents with DCD through PA, it identified potential key factors influencing intervention outcomes by thoroughly examining the sources of heterogeneity. These findings shift the research focus from “whether PA is effective” to a more clinically valuable question of “under what conditions and for which characteristics of children is it more effective?” Based on the above findings, we propose the following actionable recommendations to optimize future research and practice: (Tran et al., 2025) Future research should prioritize the critical parameter of PA intensity. High-quality RCTs should directly compare the effects of different intensity levels of PA to validate the findings of this study and provide core evidence for formulating precise exercise prescriptions (Hendrix et al., 2014). Given the methodological heterogeneity identified in this study, future research should standardize the design of the control group to enhance the interpretability of intervention effects, with a preference for activity- or time-matched alternatives when sufficient studies are available.

## 5 Research limitations

Although this meta-analysis strictly followed the PRISMA process and assessed the quality of the included studies, the following major limitations exist, and caution needs to be taken when interpreting the results:

1. Limited number of studies included: Due to the limited number of studies, only five intervention studies on MABC and four on the MABC-2 scale were included in this study, and the number of studies should be increased in the future on the basis of new RCTs in order to strengthen the robustness of the study outcomes.
2. Heterogeneity sources remain incompletely elucidated. Although subgroup and sensitivity analyses preliminarily identified PA intensity and control group design as potential sources of heterogeneity, limitations in the original study reports prevented the inclusion of other potential moderating variables (e.g., individual disability severity and comorbid conditions) in the in-depth analysis.
3. Short intervention and follow-up periods: Current evidence primarily focuses on short-to-medium-term interventions (1–4 months), with limited assessments of long-term intervention outcomes and their maintenance effects. This restricts the applicability of the conclusions to long-term rehabilitation planning.
4. Insufficient consideration of sociocultural and environmental factors: While the study encompassed diverse countries and regions with varying healthcare systems, PA promotion strategies, and cultural contexts, socioeconomic and cultural factors may influence intervention implementation and outcomes. However, this study failed to systematically evaluate and control for these variables.
5. Potential Publication and Language Bias: This study only included research published in English, potentially omitting relevant literature in other languages. This introduces language bias, affecting the comprehensiveness and generalizability of the results.

## 6 Conclusion

Based on the current limited evidence, this meta-analysis was unable to draw a definitive conclusion regarding the universal improvement effect of PA on motor coordination abilities (assessed using the MABC/MABC-2 scale) in children and adolescents with DCD. Subgroup analyses indicated that variations in the study design and intervention parameters, particularly physical activity intensity and control group structure, may contribute to inconsistent trial results. These results emphasize the importance of conducting more rigorous, well-designed, randomized controlled trials to clarify the specific effects of targeted physical activity interventions on motor coordination outcomes in this population.

## Data Availability

The original contributions presented in the study are included in the article/Supplementary material, further inquiries can be directed to the corresponding author.

## References

[ref1] Alghadier M. Alhusayni A. I. (2024). Evaluating the efficacy of gross-motor-based interventions for children with developmental coordination disorder: a systematic review. J. Clin. Med. 13:4609. doi: 10.3390/jcm13164609, PMID: 39200751 PMC11355478

[ref2] American-Psychiatric Organization (2013). Diagnostic and Statistical Manual of Mental Disorders (5th ed.). Arlington, VA: American Psychiatric Association Publishing.

[ref3] Barbosa F. V. Minatto G. Mota J. Silva K. S. de Campos W. Lopes A. S. (2016). Promoting physical activity for children and adolescents in low- and middle-income countries: an umbrella systematic review: a review on promoting physical activity in LMIC. Prev. Med. 88, 115–126. doi: 10.1016/j.ypmed.2016.03.025, PMID: 27068650

[ref4] Bonney E. Ferguson G. Smits-Engelsman B. (2017). The efficacy of two activity-based interventions in adolescents with developmental coordination disorder. Res. Dev. Disabil. 71, 223–236. doi: 10.1016/j.ridd.2017.10.013, PMID: 29055242

[ref5] Caçola P. (2016). Physical and mental health of children with developmental coordination disorder. Front. Public Health 4:224. doi: 10.3389/fpubh.2016.00224, PMID: 27822464 PMC5075567

[ref6] Cavalcante N. J. Steenbergen B. Wilson P. Zamunér A. R. Tudella E. (2020). Is Wii-based motor training better than task-specific matched training for children with developmental coordination disorder? A randomized controlled trial. Disabil Rehabil 42, 2611–2620. doi: 10.1080/09638288.2019.1572794, PMID: 30794762

[ref7] de Sousa F. M. Ordônio T. F. Santos G. Santos L. Calazans C. T. Gomes D. A. . (2020). Effects of physical exercise on neuroplasticity and brain function: a systematic review of human and animal studies. Neural Plast. 2020:8856621. doi: 10.1155/2020/8856621, PMID: 33414823 PMC7752270

[ref8] de Villiers A. Steyn N. P. Draper C. E. Fourie J. M. Barkhuizen G. Lombard C. J. . (2012). "HealthKick": formative assessment of the health environment in low-resource primary schools in the Western Cape Province of South Africa. BMC Public Health 12:794. doi: 10.1186/1471-2458-12-794, PMID: 22985326 PMC3503731

[ref9] Deng S. Li W. G. Ding J. Wu J. Zhang Y. Li F. . (2014). Understanding the mechanisms of cognitive impairments in developmental coordination disorder. Pediatr. Res. 75, 210–216. doi: 10.1038/pr.2013.192, PMID: 24192703

[ref10] Engel-Yeger B. Rosenblum S. Josman N. (2010). Movement assessment battery for children (M-ABC): establishing construct validity for Israeli children. Res. Dev. Disabil. 31, 87–96. doi: 10.1016/j.ridd.2009.08.001, PMID: 19815375

[ref11] Ferguson G. D. Jelsma D. Jelsma J. Smits-Engelsman B. C. (2013). The efficacy of two task-orientated interventions for children with developmental coordination disorder: neuromotor task training and Nintendo Wii fit training. Res. Dev. Disabil. 34, 2449–2461. doi: 10.1016/j.ridd.2013.05.007, PMID: 23747936

[ref12] Fernandes V. R. Scipi O. R. M. L. Thais M. Paulo M. P. Guimar Es T. T. An B. . (2016). Motor coordination correlates with academic achievement and cognitive function in children. Front. Psychol. 7:318. doi: 10.3389/fpsyg.2016.00318, PMID: 27014130 PMC4792186

[ref13] Fong S. Chung L. Schooling C. M. Lau E. Wong J. Bae Y. H. . (2022). Tai chi-muscle power training for children with developmental coordination disorder: a randomized controlled trial. Sci Rep 12:22078. doi: 10.1038/s41598-022-25822-x, PMID: 36543796 PMC9769475

[ref14] Fong S. S. Guo X. Liu K. P. Ki W. Y. Louie L. H. Chung R. C. . (2016). Task-specific balance training improves the sensory organisation of balance control in children with developmental coordination disorder: a randomised controlled trial. Sci Rep 6:20945. doi: 10.1038/srep20945, PMID: 26864309 PMC4750073

[ref15] Ghayour N. M. Saghaei B. Shariat A. Ingle L. Babazadeh-Zavieh S. S. Shojaei M. . (2022). Validity and reliability of the movement assessment battery second edition test in children with and without motor impairment: a prospective cohort study. Ann. Med. Surg. 77:103672. doi: 10.1016/j.amsu.2022.103672, PMID: 35638021 PMC9142614

[ref16] Griffiths A. Toovey R. Morgan P. E. Spittle A. J. (2018). Psychometric properties of gross motor assessment tools for children: a systematic review. BMJ Open 8:e21734. doi: 10.1136/bmjopen-2018-021734, PMID: 30368446 PMC6224743

[ref17] Harkness-Armstrong C. Hodson-Tole E. Wood G. Mills R. (2025). Short report on a 6-week at-home exergaming intervention to improve balance in children with developmental coordination disorder. Res. Dev. Disabil. 156:104900. doi: 10.1016/j.ridd.2024.104900, PMID: 39700647

[ref18] Hendrix C. G. Prins M. R. Dekkers H. (2014). Developmental coordination disorder and overweight and obesity in children: a systematic review. Obes. Rev. 15, 408–423. doi: 10.1111/obr.12137, PMID: 24387283

[ref19] Hillier S. McIntyre A. Plummer L. (2010). Aquatic physical therapy for children with developmental coordination disorder: a pilot randomized controlled trial. Phys Occup Ther Pedi 30, 111–124. doi: 10.3109/01942630903543575, PMID: 20367516

[ref20] Hung W. W. Pang M. Y. (2010). Effects of group-based versus individual-based exercise training on motor performance in children with developmental coordination disorder: a randomized controlled study. J. Rehabil. Med. 42, 122–128. doi: 10.2340/16501977-0496, PMID: 20140407

[ref21] Ioannidis J. P. Trikalinos T. A. (2007). An exploratory test for an excess of significant findings. Clin. Trials 4, 245–253. doi: 10.1177/1740774507079441, PMID: 17715249

[ref22] Khalki H. Lacerda D. C. Coq C. K. D. D. (2024). Early movement restriction impairs the development of sensorimotor integration, motor skills and memory in rats: towards a preclinical model of developmental coordination disorder? Eur. J. Neurosci. 60, 6830–6850. doi: 10.1111/ejn.16594, PMID: 39523702 PMC11612839

[ref23] Lee K. (2024). Enhancing motor performance and physical fitness in children with developmental coordination disorder through fundamental motor skills exercise. Healthcare-Basel 12:2142. doi: 10.3390/healthcare12212142, PMID: 39517354 PMC11545635

[ref24] Li Y. C. Wu S. K. Cairney J. Hsieh C. Y. (2011). Motor coordination and health-related physical fitness of children with developmental coordination disorder: a three-year follow-up study. Res. Dev. Disabil. 32, 2993–3002. doi: 10.1016/j.ridd.2011.04.009, PMID: 21632207

[ref25] Lloyd R. S. Oliver J. L. Faigenbaum A. D. Howard R. De Ste C. M. Williams C. A. . (2015). Long-term athletic development- part 1: a pathway for all youth. J. Strength Cond. Res. 29, 1439–1450. doi: 10.1519/JSC.0000000000000756, PMID: 25486295

[ref26] Ma A. Fong S. Guo X. Liu K. Fong D. Bae Y. H. . (2018). Adapted taekwondo training for prepubertal children with developmental coordination disorder: a randomized, controlled trial. Sci Rep 8:10330. doi: 10.1038/s41598-018-28738-7, PMID: 29985447 PMC6037761

[ref27] Macaskill P. Walter S. D. Irwig L. (2001). A comparison of methods to detect publication bias in meta-analysis. Stat. Med. 20, 641–654. doi: 10.1002/sim.698, PMID: 11223905

[ref28] Mavridis D. Salanti G. (2014). How to assess publication bias: funnel plot, trim-and-fill method and selection models. Evid-Based Ment Heal 17:30. doi: 10.1136/eb-2013-101699, PMID: 24477535 PMC13404685

[ref29] Moon J. Webster C. A. Stodden D. F. Brian A. Mulvey K. L. Beets M. . (2024). Systematic review and meta-analysis of physical activity interventions to increase elementary children's motor competence: a comprehensive school physical activity program perspective. BMC Public Health 24:826. doi: 10.1186/s12889-024-18145-1, PMID: 38491432 PMC10943790

[ref30] Page M. J. McKenzie J. E. Bossuyt P. M. Boutron I. Hoffmann T. C. Mulrow C. D. . (2021). The PRISMA 2020 statement: an updated guideline for reporting systematic reviews. Bmj-Brit Med J 372:n71. doi: 10.1136/bmj.n71, PMID: 33782057 PMC8005924

[ref31] Pranjić M. Rahman N. Kamenetskiy A. Mulligan K. Pihl S. Arnett A. B. (2023). A systematic review of behavioral and neurobiological profiles associated with coexisting attention-deficit/hyperactivity disorder and developmental coordination disorder. Neurosci Biobehav R 153:105389. doi: 10.1016/j.neubiorev.2023.105389, PMID: 37704094 PMC12042734

[ref32] Sit C. H. Yu J. J. Wong S. H. Capio C. M. Masters R. (2019). A school-based physical activity intervention for children with developmental coordination disorder: a randomized controlled trial. Res. Dev. Disabil. 89, 1–9. doi: 10.1016/j.ridd.2019.03.004, PMID: 30875607

[ref33] Song F. Sheldon T. A. Sutton A. J. Abrams K. R. Jones D. R. (2001). Methods for exploring heterogeneity in meta-analysis. Eval. Health Prof. 24, 126–151. doi: 10.1177/016327870102400203, PMID: 11523383

[ref34] Sterne J. A. C. Egger M. (2006). Regression methods to detect publication and other bias in meta-analysis. In H. R. Rothstein, A. J. Sutton, and M. Borenstein (Eds.), Publication Bias in Meta-Analysis: Prevention, Assessment and Adjustments. Chichester, England: John Wiley & Sons, Ltd, 99–110. doi: 10.1002/0470870168.ch6

[ref35] Tran H. Ho W. Chou L. Li Y. (2025). Objectively measured physical activity in children with developmental coordination disorder: a systematic review and Meta-analysis. Arch Phys Med Rehab 106, 269–279. doi: 10.1016/j.apmr.2024.06.002, PMID: 38901628

[ref36] Tsai Y. J. Hsieh S. S. Huang C. J. Hung T. M. (2021). Dose-response effects of acute aerobic exercise intensity on inhibitory control in children with attention deficit/hyperactivity disorder. Front. Hum. Neurosci. 15:617596. doi: 10.3389/fnhum.2021.617596, PMID: 34220467 PMC8249764

[ref37] Valentine J. C. Pigott T. D. Rothstein H. R. (2011). How many studies do you need? A primer on statistical power for meta-analysis. Qual. Cont. Appl. Statistics 35, 215–247. doi: 10.3102/1076998609346961, PMID: 38293548

[ref38] van der Hoek F. D. Stuive I. Reinders-Messelink H. A. Holty L. de Blécourt A. C. Maathuis C. G. . (2012). Health-related physical fitness in Dutch children with developmental coordination disorder. J. Dev. Behav. Pediatr. 33, 649–655. doi: 10.1097/DBP.0b013e3182653c50, PMID: 23027139

[ref39] Verbecque E. Denysschen M. Coetzee D. Valtr L. Bonney E. Smits-Engelsman B. (2025). Which items of the movement assessment battery for children are most sensitive for identifying children with probable developmental coordination disorder? Results from a large-scale study. Res. Dev. Disabil. 157:104904. doi: 10.1016/j.ridd.2024.104904, PMID: 39787776

[ref40] Wilson P. H. Adams I. L. Caeyenberghs K. Thomas P. Smits-Engelsman B. Steenbergen B. (2016). Motor imagery training enhances motor skill in children with DCD: a replication study. Res. Dev. Disabil. 57, 54–62. doi: 10.1016/j.ridd.2016.06.014, PMID: 27388492

[ref41] Yu J. J. Burnett A. F. Sit C. H. (2018). Motor skill interventions in children with developmental coordination disorder: a systematic review and Meta-analysis. Arch Phys Med Rehab 99, 2076–2099. doi: 10.1016/j.apmr.2017.12.009, PMID: 29329670

