# Supplementary material for: Intervention effect of physical activity on motor coordination in children and adolescents with developmental coordination disorder: a meta-analysis
Source: Front Psychol. 2025 Nov 19;16:1630439. doi: 10.3389/fpsyg.2025.1630439 (PMC12672234; doi:10.3389/fpsyg.2025.1630439)
Supplement: Supplementary file 1 [file Data_Sheet_1.docx]

**SUPPLEMENTAL MATERIAL**

**Table S1.**Egger's test for the effect of physical activity on motor coordination in children and adolescents with developmental coordination disorder.

**Figure S1.**Subgroup analysis of MABC outcome measures.

**Figure S2.**Subgroup analysis of MABC-2 outcome measures.

**Figure S3.**Egger tests of exercise intervention on MABC outcome measures in children and adolescents with developmental coordination disorder.

**Figure S4.**Egger tests of exercise intervention on MABC-2 outcome measures in children and adolescents with developmental coordination disorder.

**Figure S5.**Sensitivity analysis results of physical activity on MABC-2 outcome measures.

**Table S1**EEgger's test for the effect of physical activity on motor coordination in children and adolescents with developmental coordination disorder.

| **Outcome indicators** | ***N*** | ***t*** value | ***P*** value | **[95%CI]** |
| --- | --- | --- | --- | --- |
| MABC | 5 | -0.94 | 0.416 | [-3.936,2.141] |
| MABC-2 | 6 | 1.25 | 0.278 | [-11.941,31.621] |

**Figure S1.**Subgroup analysis of MABC outcome measures.

**
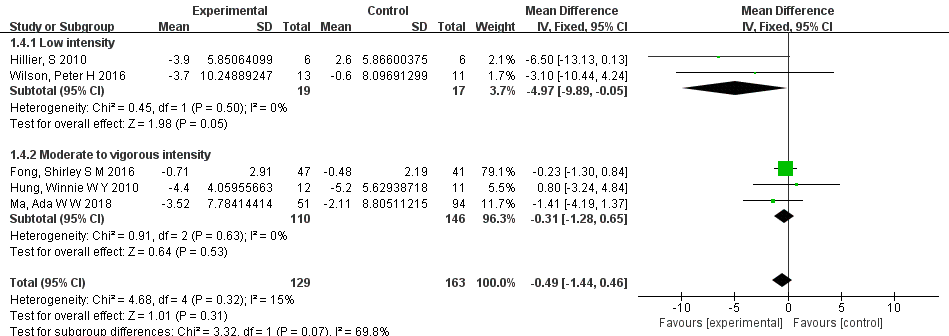
**

**(a)Intensity of physical activity**

**
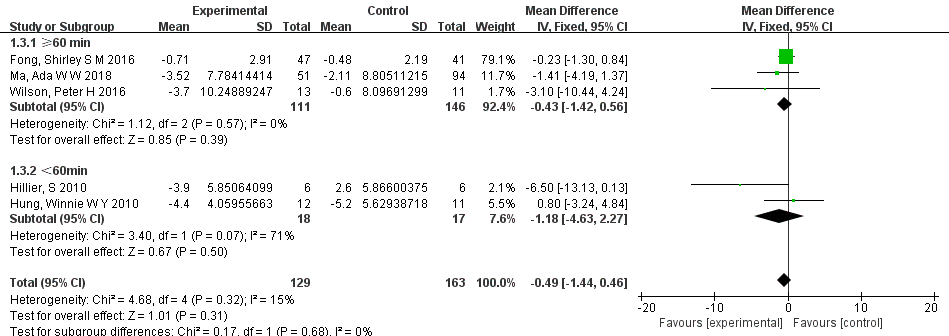
**

**(b)Single exercise duration**

**
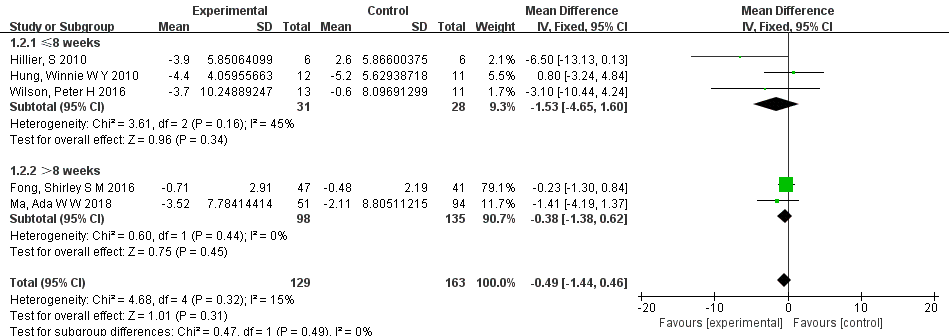
**

**(c)Total intervention period**

**Figure S2.**Subgroup analysis of MABC-2 outcome measures.


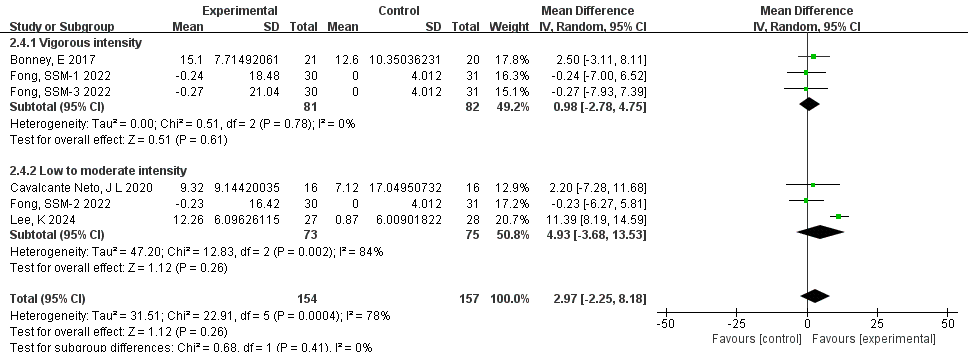


**(a)Intensity of exercise**

**
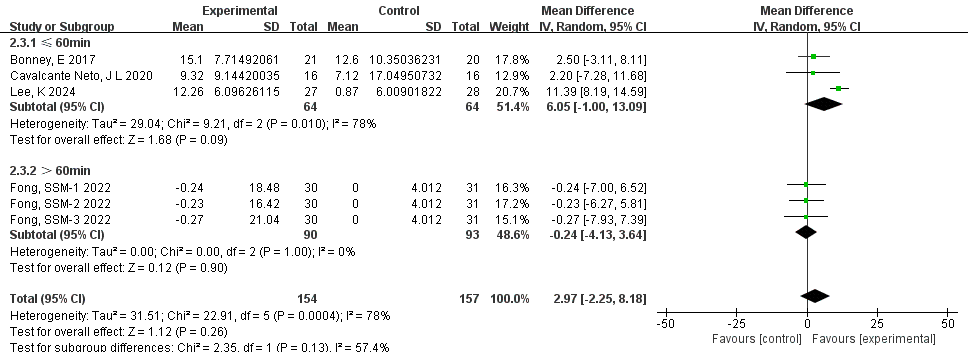
**

**(b)Single exercise duration**

**
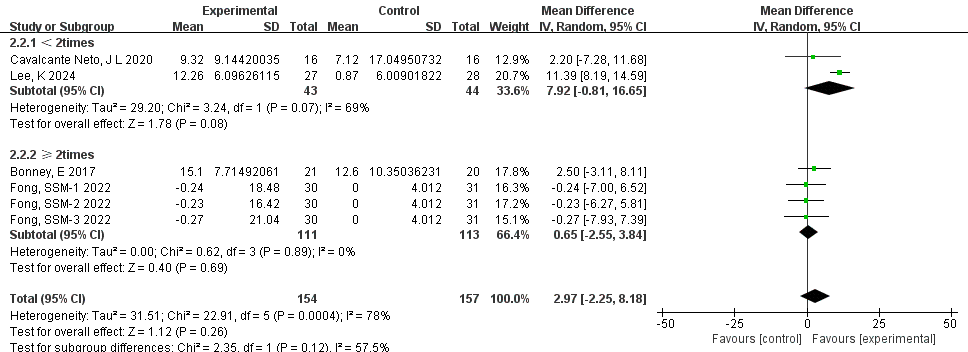
**

**(c)Weekly exercise frequency**

**Figure S3.**Egger tests of exercise intervention on MABC outcome measures in children and adolescents with developmental coordination disorder.

**(a)**

**(b)**

**Figure S4.**Egger tests of exercise intervention on MABC-2 outcome measures in children and adolescents with developmental coordination disorder.

**(a)**

**(b)**

**Figure S5.**Sensitivity analysis results of physical activity on MABC-2 outcome measures.

**(a)**

**(b)**
